# Supplementary material for: Prevalence of active tuberculosis disease among healthcare workers and support staff in healthcare settings of the Amhara region, Ethiopia
Source: PLoS One. 2021 Jun 11;16(6):e0253177. doi: 10.1371/journal.pone.0253177 (PMC8195404; doi:10.1371/journal.pone.0253177)
Supplement: Attachment — Submitted filename: Plos one 2021 March Comments.docx [file pone.0253177.s002.docx]

The Editor,

Plosone,

Dear Sir,

Thanks for asking me to review the manuscript titled: “The Prevalence of active tuberculosis disease among healthcare workers and support staff in healthcare settings of the Amhara region, Ethiopia.” (PONE-D-21-03747R1) for the second time.

The authors had done the corrections but the manuscript still need to be edited for grammatical errors.

In addition, I suggest that the variables in table one should be listed in descending other;

Support staff:

Cleaner	58(20.0)

Data clerk 51(17.6)

Cashier 42(14.5)

HCWs:

Nurse 122 (42.1)

Laboratory staff 50(17.2)

Pharmacy staff 34(11.7)

Regards

DR. Kuyinu Yetunde, FMCPH

1. Recommendation: **Minor Revision.**
2. Is the manuscript technically sound, and do the data support the conclusions? **Yes**
3. Has the statistical analysis been performed appropriately and rigorously? **Yes**

4. Does the manuscript adhere to the PLOS Data Policy? **Yes**
5. Is the manuscript presented in an intelligible fashion and written in Standard English? **Yes**
6. Review Comments to the Author (minimum 100 characters)
7. PLOS authors have the option to publish the peer review history of their article (https://journals.plos.org/plosone/s/editorial-and-peer-review-process#loc-peer-review-history). If published, this will include your full peer review and any attached files. Do you want your identity to be public for this peer review? **No**
7a. If you answered "Yes" above, that you would like your identity to be public, please provide your full name, as it should appear on the published peer review. Please do not sign the review on behalf of another person.

Confidential to Editor:
1. Do you have any potentially competing interests? **No**
2. Did you receive any assistance in preparing this review (e.g. from a post-doc or graduate student)? **No**
