# Supplementary material for: Prevalence of active tuberculosis disease among healthcare workers and support staff in healthcare settings of the Amhara region, Ethiopia
Source: PLoS One. 2021 Jun 11;16(6):e0253177. doi: 10.1371/journal.pone.0253177 (PMC8195404; doi:10.1371/journal.pone.0253177)
Supplement: Attachment — Submitted filename: point by point.docx [file pone.0253177.s003.docx]

Dear editor, thank you for giving us the opportunity to revise our manuscript and we also thank the reviewers for comments and critics helping us to improve our paper. The following are point by point responses.

The authors had done the corrections but the manuscript still need to be edited for grammatical errors.

Response: thank you, grammatical errors have been corrected throughout the whole manuscript.

In addition, I suggest that the variables in table one should be listed in descending other;

Support staff:

Cleaner	58(20.0)

Data clerk 51(17.6)

Cashier 42(14.5)

HCWs:

Nurse 122 (42.1)

Laboratory staff 50(17.2)

Pharmacy staff 34(11.7)

Response: thank you for the valuable comment. Now, table one has been updated according to the reviewer’s advice
